# C/EBPα Epigenetically Modulates *TFF1* Expression *via* mC-6 Methylation in the Jejunum Inflammation Induced by a Porcine *Coronavirus*

**DOI:** 10.3389/fimmu.2022.881289

**Published:** 2022-05-25

**Authors:** Huan Qu, Qiufang Zong, Haifei Wang, Shenglong Wu, Demin Cai, Wenbin Bao

**Affiliations:** ^1^ College of Animal Science and Technology, Yangzhou University, Yangzhou, China; ^2^ Joint International Research Laboratory of Agriculture and Agri-Product Safety, Yangzhou University, Yangzhou, China

**Keywords:** TFF1, DNA methylation, C/EBPα, porcine epidemic diarrhea virus, intestinal epithelial barrier, coronavirus

## Abstract

Porcine epidemic diarrhea virus (PEDV) is an emerging coronavirus which causes acute diarrhea and destroys gastrointestinal barrier function in neonatal pigs. Trefoil factor 1 (TFF1) is a protective peptide for maintaining the integrity of gastrointestinal mucosa and reducing intestinal inflammation. However, its role in protecting intestinal epithelium against PEDV infection is still unclear. In this study, we discovered that TFF1 expression was activated in the jejunum of pigs with PEDV infection and TFF1 is required for the growth of porcine intestinal epithelial cells. For instance, inhibited cell proliferation and cell arrest were observed when *TFF1* is genetically knocked-out using CRISPR-Cas9. Additionally, *TFF1* depletion increased viral copy number and PEDV titer, along with the elevated genes involved in antiviral and inflammatory cytokines. The decreased *TFF1* mRNA expression is in line with hypermethylation on the gene promoter. Notably, the strong interactions of protein-DNA complexes containing CCAAT motif significantly increased C/EBPα accessibility, whereas hypermethylation of mC-6 loci decreased C/EBPα binding occupancies in *TFF1* promoter. Overall, our findings show that PEDV triggers the C/EBPα-mediated epigenetic regulation of *TFF1* in intestine epithelium and facilitates host resistance to PEDV and other *Coronavirus* infections.

## Introduction

Porcine epidemic diarrhea is a highly contagious enteric disease caused by porcine epidemic diarrhea virus (PEDV) which is characterized by intestinal inflammation, watery diarrhea, vomiting, or dehydration. All ages and breeds of pigs are susceptible to PEDV, whereas it is most harmful to neonatal piglets (1). PEDV causes an 80-100% fatality rate in piglets, and more than 10% of the pig population is wiped out throughout the world (2). PEDV is a single-stranded positive-sense RNA virus which is classified into the genus α-*Coronavirus* of the *Coronaviridae* family. It contains four structural proteins: spike (S), envelope (E), matrix (M), and nucleocapsid (N) (3, 4). This virus infection is usually transmitted by the fecal-oral route or air from the nasal cavity to the intestinal mucosa and replicates principally in the intestinal epithelial cells (especially in the jejunum and ileum) (5, 6). Given that the functional integrity of intestinal epithelial barrier constitutes the main defense against pathogens invasion, its physiological homeostasis is critical for intestinal against PEDV infection.

The trefoil factor family (TFFs) is a group of protein polypeptides produced mainly by gastrointestinal mucus-secreting or mucin-secreting cells. TFFs are widely distributed in vertebrates and highly conserved in evolution, suggesting that they may be a class of proteins with important physiological functions (7, 8). TFF1 (also known as pS2), as a member of the TFFs, is a cysteine-rich secretory protein with the function of gastrointestinal protection (9, 10), mucosal defense, and injury healing (11–13). When co-expressed with the mucin MUC5AC, TFF1 works for mucosal protection by forming a stable mucus gel layer together with mucin. During the extracellular pathogen invasion, the anti-apoptotic activity and the epithelial cell migration are coordinately controlled by TFF1 to improve injury repair and immune response (14–16). Importantly, the *TFF1* gene shows the tissue-specific expression pattern and is closely related to the ability of injury healing, activating the host’s resistance to pathogens infection (17, 18). Previously, we have demonstrated a 28.8-fold increase in *TFF1* gene expression by transcriptome analysis in PEDV-infected jejunum (19). In this study, we aim to identify whether *TFF1* is a potential target to benefit gut function in response to PEDV infection.

In recent years, the development of epigenetics provides new insights to solve the genetic mechanism of diseases. Among them, methylation of CpG residues is a key epigenetic modification of eukaryotic DNA which provides a stable gene silencing and is a major factor responsible for the suppression of gene expression (20). Importantly, heterochromatin domains are determined, in part, by methylation of cytosines at the 5^th^ position of the pyrimidine ring. Indeed, previous reports revealed that the *TFF1* gene expression is vulnerable to DNA methylation pattern (21, 22) and it has been documented that hypermethylation of *TFF1* promoter triggers the gene downregulation in intestinal metaplasia and intestinal-type human cell lines (22, 23). So, this may be attributed to the impaired transcription factor like C/EBPα and/or HIF-1α modulation induced by methylation events (24, 25). Thus, this study aims to understand the *TFF1* action of inflammatory inhibition in the PEDV-infected jejunum and the underlying mechanisms. Our findings exhibit that *TFF1* is a resistant factor to a porcine *coronavirus* infection by the regulation of C/EBPα and DNA methylation.

## Materials and Methods

### Ethics Statement

The animal study proposal was approved by the Institutional Animal Care and Use Committee (IACUC) of the Yangzhou University Animal Experiments Ethics Committee (permit number: SYXK (Su) IACUC 2012-0029). All experimental methods were conducted in accordance with the relevant guidelines for the Administration of Affairs Concerning Experimental Animals approved by the State Council of the People’s Republic of China.

### Animal Experiments and Samples Collection

Four 7-day-old ternaries crossbred piglets (Duroc*Landrace*Yorkshire) were infected with PEDV featured with typical clinical symptoms of porcine epidemic diarrhea. In addition, four normal piglets were selected as the control. The herd of pigs was detected for PCR against PEDV, TGEV, PDCoV, and PoRV. All the animals were raised under the same conditions and humanely sacrificed by intravenous injection of pentobarbital sodium. After the sacrifice, duodenum, jejunum, and ileum samples were snap frozen immediately in liquid nitrogen for the subsequent experiments.

### Etiology Identification

Jejunal contents were collected and diluted in 500 μL phosphate-buffered saline (PBS) buffer, then freeze-thawed repeatedly before the centrifugation to collect the supernatant containing viruses. The supernatants were added to the tubes containing TRIzol reagent (Takara Biotech, Dalian, China) and vortexed according to the manufacturer’s instruction. The cDNA was amplified with PrimeScript RT-PCR kit (Vazyme Biotech, Nanjing, China) to identify the infections of viruses, including PEDV, TGEV, PDCoV and PoRV. The primers are listed in **Table 1**.

**Table 1:** Primers and sequence information for pathogen identification.

Target genes	GenBank Accession number	Primers sequences (5’-3’)	Length (bp)	Temperature (°C)
*PEDV(M)*	AF353511.1	F: AGGTCTGCATTCCAGTGCTT R: GGACATAGAAAGCCCAACCA	216	60
*TGEV(S)*	FJ755618	F: CCAAACAGCCGTTATTAGTTA R: AGTGACACCACCCGTTGT	218	60
*PDCoV(N)*	JQ065043	F: ATGGCTACTGGCTGCGTTAC R: GCGTTTCCTGGGCTGATT	383	60
*PoRV(VP6)*	FJ807867	F: CAAACGGGAGGAATAGGAA R: CACTCTTGGGAAACTGAACC	527	60

### Histological Analysis

Duodenum, jejunum, and ileum were fixed in 4% paraformaldehyde solution for 24 hours. Tissues were embedded in paraffin and cut into 5 μm pieces. For hematoxylin-eosin (HE) staining, the paraffin sections were conducted according to the routine procedure. The mounted slides were observed under Olympus iX53 (Tokyo, Japan) light microscope and the photographs were taken using Motic Image Advanced 3.2 Analysis System (Motic China group Co., Ltd., Xiamen, China).

### Cell Culture

Porcine intestinal epithelial cells (IPEC-J2) were kindly provided by the University of Pennsylvania. Vero kidney cell line (Vero CCL-81) were purchased from ATCC. IPEC-J2 and Vero cells were cultured in high-glucose Dulbecco’s modified Eagle’s medium (DMEM, Gibco, NY, USA) supplemented with 10% fetal bovine serum (FBS, Gibco, NY, USA) and 100 μg/mL penicillin/streptomycin (Solarbio, Beijing, China) in a humidified atmosphere containing 5% CO_2_ at 37°C. Cells were seeded in tissue culture flasks (Costar, Corning, MA, USA).

### Virus Infection *In Vitro*

PEDV strains used in this study were the classic strain CV777, which was propagated and titrated in FBS-free DMEM medium supplemented with 8 μg/mL trypsin (Sigma-Aldrich, MO, USA) in Vero CCL-81 cells. Cells were infected with PEDV at MOI of 0.1 for 2 hours at 37°C (26). After 2 hours, the unattached virus inoculum was removed. Then, the cells were washed three times with cold PBS buffer and maintained in DMEM supplemented with 2% FBS. The infected cells and supernatants were collected after the indicated period and titrated according to TCID_50_ protocol. The viral titers were calculated using the Spearman-Karber equation. The PEDV genome copy number was calculated by the standard curve equation of CV777 well-established previously (26): y = −3.3354lg(x) + 37.832 (where y represents the number of Ct cycles, x represents the logarithm of the virus copy number based on the base 10).

### CRISPR/Cas9 sgRNA Genomic Editing

To deplete *TFF1* gene, three different single guide RNAs (sgRNAs) were designed using an CRISPR-Cas9 Design online software. Oligo nucleotides sequences of the sgRNAs were synthesized and cloned into the *Bbs*I digested linearized pGK1.2 (EGFP + Puro^r^) vector (Genloci Biotech, Nanjing, China) following the pGK1.2 oligo-cloning protocol. The primers are listed in **Table 2**. Colony PCR was performed to validate the sgRNA sequences using the VSP forward primer 5′-CATATGCTTACCGTAACTTGAAAG-3′ and three primer sets: primer set-1: (5’-GCCACAGGATTGAAGCACCA-3’), primer set-2: (5’-GTTGTCTGTGGGGTCATCAA-3’), primer set-3: (5’-GTGGGGTCATCAACGGCCAC-3’) as the reverse primers, respectively. The amplicons were performed by PCR-sequencing (Sangon Biotech, Shanghai, China) and the positive recombinant vectors were named as sgRNA1, sgRNA2 and sgRNA3, respectively. Subsequently, cells pool sequencing verified the efficiency of sgRNAs mixture. The primer used for cloning was as follows: *TFF1-sgRNA* forward primer (5’-CTTGGCCGGTACACTTTCAG-3’) and reverse primer (5’-CTTCACAGGTCCGTGGTTAGA-3’). IPEC-J2 were transfected with sgRNA1, sgRNA2 and sgRNA3 in 6-well tissue culture plates at a density of 2 × 10^5^/mL. The culture medium with 3 μg/mL puromycin was added to the cells 72 hours later. For another 72 hours, positive monoclonal cells were harvested.

**Table 2:** CRISPR/Cas9 sgRNA oligo sequences.

Oligo Name	Forward sequences (5’-3’)	Reverse sequences (5’-3’)
sgRNA1	F: CACCGCCACAGGATTGAAGCACCA	R: AAACTGGTGCTTCAATCCTGTGGC
sgRNA2	F: CACCGTTGTCTGTGGGGTCATCAA	R: AAACTTGATGACCCCACAGACAAC
sgRNA3	F: CACCGTGGGGTCATCAACGGCCAC	R: AAACGTGGCCGTTGATGACCCCAC

### Overexpression Plasmid Construction and Cells Transfection

For *TFF1* and *C/EBP*α overexpression, the full-length CDS of *TFF1* and *C/EBP*α were amplified and cloned into a pcDNA3.1 vector (Invitrogen, CA, USA) with T4 DNA ligase for ligation by *Nhe*I and *Hind*III restriction sites, respectively. The recombinant overexpression vectors were defined as *TFF1*-oe and *C/EBP*α-oe, respectively. Subsequently, each of the overexpression vectors were transfected into IPEC-J2 using Lipofectamine™ 2000 (Invitrogen, CA, USA) following the manufacturer’s protocol. At 36 hours after transfection, the cells were passaged into the appropriate medium containing 400 µg/mL G418. The screening was conducted over 7 days and the stable cell lines constitute polyclonal pools of cells.

### siRNA Transfection

Small interfering RNAs (siRNAs) against *C/EBP*α mRNA sequences (si-*C/EBP*α-1, si-*C/EBP*α-2, and si-*C/EBP*α-3) of *sus scrofa* and negative control (si-*C/EBP*α-NC) were synthesized from GenePharma Corporation (Shanghai, China). The siRNAs and negative control were transfected into IPEC-J2 using Lipofectamine™ 2000 (Invitrogen, CA, USA) following the manufacturer’s instruction.

### Cell Proliferation Assay

To assess cell viability, cells were seeded in 96-well plates with 5 × 10^3^/well and incubated with PEDV at the indicated time. Then 10 μL cell counting kit-8 solution (CCK8, Dojindo Molecular Technologies Inc., Kumamoto, Japan) was added to each well followed by the incubation at 37°C for 2 hours. After the incubation, an absorbance at 450 nm was measured using a Multimode Microplate Reader (Spark™ 10M, Tecan GmbH, Austria). Caspase-3/7 activity was measured using a luminescence-based assay with the luminescent Caspase-Glo 3/7 assay kit (Promega Corporation, MA, USA) according to the manufacturer’s instruction. Each experiment was repeated three times and performed in triplicate.

### Flow Cytometry Analysis

Cells were seeded in 6-well plates and incubated with the indicated doses of PEDV. The apoptotic cells were detected by Annexin V-FITC/PI apoptosis analysis kit (Solarbio, Beijing, China) according to the manufacturer’s guide. The samples were analyzed by CytoFLEX flow cytometer (Beckman Coulter, CA, USA). For cell cycle detection, *TFF1*-depletion cells were seeded in tissue culture flask at a density of 2 × 10^6^/mL. Cells were then washed with cold PBS and fixed overnight with cold 70% ethanol. The fixed cells were washed with PBS and stained with 1 mL PI staining reagent (50 mg/mL propidium iodide and 1 mg/mL RNase A in 1 mL of sodium citrate buffer) for 30 min at 37°C in a light-proof manner. Then the cell cycle profiles were analyzed by FACScan flow cytometer (Becton Dickinson, CA, USA). The percentage of cells at G0/G1, S and G2/M phases were calculated using Modifit software.

### Indirect-Immunofluorescence Assay (IFA)

Cells were seeded on coverslips in 12-well tissue culture plates and infected with PEDV for 2 hours, rinsed, then incubated at 37°C. After 48 hours of PEDV infection, the cells were fixed in 4% paraformaldehyde for 15 min and permeabilized by 0.1% Triton X-100 in PBS for 10 min at RT. The cells were blocked with 5% bovine serum albumin (BSA) in PBS for 30 min at RT and then incubated with the anti-PEDV antibody PEDV N MAb (Medgene, SD-1-5, 1:500) overnight at 4°C. After washing thrice with PBS, the cells were incubated with a fluorochrome-conjugated secondary antibody (HuaBio, HA1015, 1:100) for 30 min at RT. The cell nuclei were stained with DAPI (1 μg/mL) for 5 min. The coverslips were mounted on microscope slides for the visualization under a fluorescence microscope (Olympus, Tokyo, Japan). Images were processed using ImageJ software.

### The Inflammatory Cytokines and Antiviral Genes mRNA Expression

Cells were treated with PEDV at the indicated time as above. RT-qPCR was performed to detect the mRNA expression of inflammatory cytokines (*IL-2*, *IL-6*, *IL-8*, *IL-12*, *IL-α*, *IL-β*, *IFN-α*, *IFN-β*, *TNF-α*) and antiviral genes (*Mx1*, *Mx2*, *RIG*). The primers are listed as shown in **Table 3**.

**Table 3:** Primers of inflammatory cytokines and antiviral genes.

Target genes	GenBank Accession number	Primer sequences (5’-3’)	Length (bp)
*TNF-α*	JF831365.1	F: CCTACTGCACTTCGAGGTTATC R: GCATACCCACTCTGCCATT	158
*IL-6*	NM_001252429.1	F: ATCTGGGTTCAATCAGGAGACCT R: ATTTGTGGTGGGGTTAGGGG	208
*IL-8*	NM_213867.1	F: CCACACCTTTCCACCCCAAA R: TTGTTGCTTCTCAGTTCTCTTCA	179
*IL-12*	NM_213993.1	F: CAGGCCCAGGAATGTTCAAA R: CGTGGCTAGTTCAAGTGGTAAG	166
*IFN-α*	KF414740.1	F: TTCTGCACTGGACTGGATC R: TCTGTGGAAGTATTTCCTCACAG	103
*IFN-β*	JN391525.1	F: GCTAACAAGTGCATCCTCCAAA R: CCAGGAGCTTCTGACATGCCA	124
*IL-α*	NM_214029.1	F:ACCTGGATGAGGCAGTGAAAT R:ATGGGCGGCTGATTTGAAGT	236
*IL-β*	XM_005670069.3	F:AAGAAAGTGCGGCGGAAAGTA R:CCACAGAAGTCCCATCCTTAC	177
*IL-2*	XM_003358973.3	F: GTTGCATTGCACTAACCCTT R: TGGCTCCAGTTGTTTCTTTG	86
*Mx1*	NM_214061.2	F: GTCATCGGGGACCAGAGTTC R: TCCCGGTAACTGACTTTGCC	164
*Mx2*	NM_001097416.1	F: CCAGAGGCAGCGGAATCAT R: TTTGCGTATTTCCCGCTCCA	143
*RIG-1*	NM_213804.2	F: AAGAAGAGTACCACTTAAACCCAG R: ATGCCTTCATCTGCCACCGA	256

### Reverse Transcription and Quantitative PCR (RT-qPCR)

Total RNA was isolated from cells and intestinal samples using the TRIzol reagent (Takara Biotech, Dalian, China) following the manufacturer’s protocol. RT-qPCR amplification was performed using an ABI 7500 Fast Real-Time Quantitative PCR System (Applied Biosystems, Foster City, CA, USA) and relative gene expression was calculated using the 2^−ΔΔCt^ method (27). The housekeeping gene *GAPDH* was used as an internal control for each experiment. The experiments were performed at least three times with data presented as means values ± SD. The primers are shown in **Table 4**.

**Table 4:** RT-qPCR primers and sequence information.

Target genes	GenBank Accession number	Primer sequences (5’-3’)	Length (bp)	Temperature (°C)
*TFF1*	XM_003358973.3	F: AAGGTGATCGGTGTCCTGGT R: CCGGAGAAACCACAGTTCA	117	60
*GAPDH*	AF017079.1	F: ACATCATCCCTGCTTCTACTGG R: CTCGGACGCCTGCTTCAC	187	60

### Western Blotting Analysis

Intestinal tissues and cells were lysed in radioimmunoprecipitation assay (RIPA) buffer that contains proteinase inhibitor cocktail on ice for 20 min. The supernatant was gathered, centrifuged at 12,000 g for 20 min, and the protein concentration was determined using the BCA Protein Assay Kit (CWBiotech, Beijing, China). Proteins were separated in SDS-PAGE and transferred to a PVDF membrane (Millipore, MA, USA). The membranes were blocked with 5% skimming milk in Tris-buffered saline-Tween (TBST) for 1 hour, then incubated at 4°C overnight with the primary antibodies [anti-TFF1 (LifeSpan BioScience, ls-c312842, 1:1000), anti-CDK4 (Proteintech Ltd, 11026-1-AP, 1:1000), anti-PCNA (Proteintech Ltd, 10205-2-AP, 1:1000), anti-Bax (HuaBio, ET1603-34, 1:1000), anti-Cleaved Caspase 3 (Cell Signaling Technology, #9664, 1:500), anti-PEDV-N (Youlong-Bio, DA0110,1:1000), anti-β-Actin (CWBIO, CW0096M, 1:1000) and anti-GAPDH (Proteintech Ltd, 10494-1-AP, 1:1000)], respectively. The membranes were rinsed and incubated with the HRP-conjugated secondary antibodies [goat anti-Rabbit IgG antibody (Abcam, ab205718, 1:20000) and anti-Mouse IgG antibody (HuaBio, HA1006, 1:10000)] for 1 hour at RT. The membranes were visualized with an Enhanced Chemiluminescent Detection kit (ThermoFisher Scientific, MA, USA) using the FluorChem FC3 Chemilumilescent system (ProteinSimple Ltd, CA, USA). The relative integrated density was measured and normalized against GAPDH or β-Actin expression. The experiments were performed at least three times with data presented as means values ± SD.

### DNA Isolation and Bisulfite Treatment

The genomic DNA was extracted from jejunum in diarrheic and normal piglets using TIANamp Genomic DNA Kit (Tiangen Biotech, Beijing, China). The extracted DNA was bisulfite conversed using EZ DNA Methylation-Gold Kit (Zymo Research, CA, USA). The conversion DNA was performed PCR amplification using ZymoTaq Premix, with the primers designed for amplification presented in **Table 5**. The amplification system contains 2 μL DNA, 12.5 μL ZYMO Taq Premix, 1 μL forward primer and 1 μL reverse primer (10 pmol/μL) and RNase-free H_2_O 8.5 μL. The following PCR reaction was performed to 95°C for 10 min, followed by 40 cycles with each cycle consisting of 95°C for 30 s, and 54°C for 30 s, extension at 72°C for 30 s, and a final 5 min extension at 72°C. The PCR products were purified by TIANquick Midi purification kit (Tiangen Biotech, Beijing, China) and ligated into pMD19-T vector (Takara Biotech, Dalian, China) at 16°C overnight and transformed into *Escherichia coli* DH-5α competent cells. More than 15 positive clones from each sample were randomly picked up for bisulfite-sequencing. The methylation status at each CpG site was aligned using QUMA online software.

**Table 5:** The primers of CpG island and BSP-PCR primers in *TFF1* promoter.

Target genes	Primers sequences (5’-3’)	Length (bp)	Temperature (°C)
*TFF1*-CpG	F: GGACTAGTACCCTCACCTGCTCTGTCCC R: CATGCCATGGCCCCCAGCCCTTCATTCCAA	334	63
*TFF1*-BSP	F: AAAGGATTAGGTTGGGTTAAGGAT R: AACTTACAAAAAATCCCTCCCTTAT	244	60

### CpG Methyl Transferase *M.Sss*I and 5’-Aza-2’-Deoxycytidine Treatment

The CpG islands in the promoter region of *TFF1* were predicted by MethPrimer website. The parameters were set as follows: CpG islands length greater than 200 bp, GC content greater than 50%, and CpG o/e greater than 0.6. For the reporter-gene assays of methylation on *TFF1* promoter, the CpG island fragment of *TFF1* promoter (−145 to −478 bp) was ligated to the pGL3-basic vector. The vectors were treated with *M*.*Sss*I (CpG) methyl transferase (New England Biolabs, Beijing, China) and transfected into IPEC-J2. Luciferase assays were performed 36 hours after transfection. The primer for CpG islands amplification is presented in **Table 5**. IPEC-J2 were treated with concentration of 0.1 μM 5’-Aza-2′-deoxycytidine (5’-Aza-2’-dC, Sigma-Aldrich, MO, USA) for 72 hours. Then the cells were harvested for mRNA expression analysis.

### Chromatin Immunoprecipitation (ChIP) Assay

ChIP assay was performed using the Pierce Agarose ChIP Kit (ThermoFisher Scientific, MA, USA) following the manufacturer’s instructions. An amount of 80 mg sheared jejunum sample was minced and crosslinked in formaldehyde. After the chromatin digested with micrococcal nuclease, the crude chromatin fragments were immunoprecipitated with the specific anti-C/EBPα antibody (Santa Cruz, sc-365318, 1:500) overnight at 4°C and then incubated with protein-G magnetic beads. Next, the enriched chromatin was purified for ChIP-PCR amplification and the amplified products were examined with 2.0% agarose gel electrophoresis. The primer is presented as follows: forward primer (5’-CGCAGCATCTCTGCTGTGAA-3’) and reverse primer (5’-ACCCTCCCGCTAAGTCAACA-3’).

### Electrophoretic Mobility Shift Assay (EMSA)

The probes for electrophoretic mobility shift assay (EMSA) were as follows: the wild-type probe (5′-ACCGCGCTGGCGCAGCATCT-3′) and the mutant-type probe (5′-ACCGCATCAATATGACATCT-3′). Briefly, an oligonucleotide containing the C/EBPα motif and its complementary sequences were synthesized and then annealed to double-stranded structures. The wild-type probe was treated by methyltransferase *M.Sss*I (New England Biolabs, Beijing, China). Total nuclear proteins were extracted from trypsinized monolayers of IPEC-J2 by a Nuclear and Cytoplasmic Protein Extraction Kit (Beyotime, Nanjing, China). The double-stranded oligonucleotide was 5′ end-labeled with biotin-dUTP. For each binding reaction, 2 μg labeled probes, 2 μg nuclear extract, 4 μg poly (dI–dC), 10 μL binding buffer [20 mM Tris-HCl (pH 7.6), 50 mM KCl, 1 mM DTT, 0.5 mM EDTA, and 10% glycerol] were incubated at 25°C for 30 min. Then the protein–DNA complexes were separated on non-denaturing 6% polyacrylamide gels with precooled 0.5 × TBE buffer. For the super-shift assay, 1 μL anti-C/EBPα antibody (Santa Cruz, sc-365318, 1:500) was added to EMSA reaction mixtures and incubated for 60 min at 4°C prior to the labeled probe addition. Then, the protein–DNA complexes were transferred to nylon membrane at 60 v for 1 hour and autoradiography of the dried gel was detected immediately after UV cross-linking.

### Construction of Truncated Sequence Plasmids

The *TFF1* promoter sequences were analyzed by Alibaba2 online software to predict the potential transcription factor binding sites (TFBS). According to the core promoter prediction of BDGP online software, the PCR purified products were digested with *Kpn*I and *Xho*I and ligated to the pGL3-basic luciferase reporter vector. Four plasmids with truncated promoters were constructed by inserting the DNA sequences of the *TFF1* promoter ranging from −1200 to −1. The truncated recombinant vectors were defined as pGL3-control (−200 to −1), pGL3-*TFF1*-p1 (−360 to −1), pGL3-*TFF1*-p2 (−500 to −1) and pGL3-*TFF1*-p3 (−1200 to −1).

### Dual-Luciferase Reporter Assay

For the reporter-gene assays, pGL3-*TFF1*-wt was constructed by inserting the DNA fragments of the *TFF1* promoter from −360 to −200 into pGL3-basic vector. The mutant form pGL3-*TFF1*-mut contains the sequences mutated from GCTGGCGCAG to ATCAATATGA. In addition, the sequences GCTGGCGCAG enrolled in pGL3-*TFF1*-wt was deleted and named as pGL3-*TFF1*-del. The IPEC-J2 were co-transfected with *C/EBPα*-oe and wild type, mutant or deletion forms of *TFF1* promoter reporter constructs. The renilla was co-transfected for normalization. After 36 hours post-transfection, luciferase activity was analyzed with a Dual-Luciferase Assay system (Promega Corporation, Madison, USA) on a luminometer according to the manufacturer’s instruction. The relative fluorescence intensity was calculated as Firefly-Luc (Ff)/Renilla-Luc (Rn). All the transfections were performed in sextuplicate and each experiment was repeated at least three times.

### Statistical Analysis

Statistical analysis was performed by GraphPad Prism 8.0 software. All statistical details of experiments were included in the figure legends. All experiments were repeated at least three times and the results were shown as mean values ± SD.

## Results

### *TFF1* Is Associated With the Host Responses of PEDV Infection

**Figure 1:**
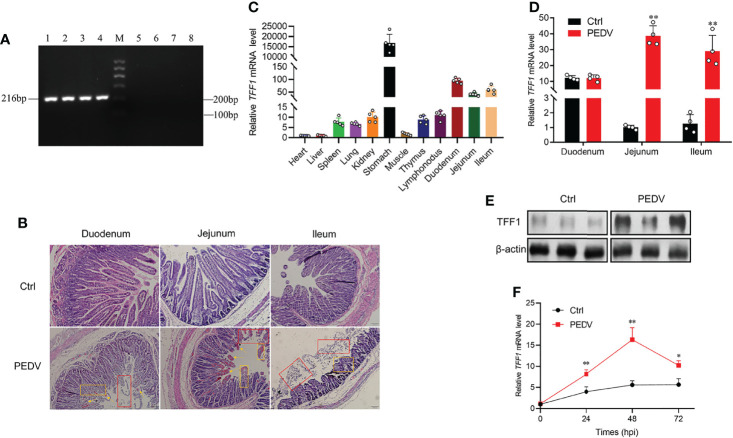
*TFF1* is a key candidate responsible for PEDV infection. **(A)** RT-qPCR detection of *M* gene expression by 2.0% agarose gel electrophoresis. Lanes 1-4, intestinal contents of diarrhea piglets; Lane M, Marker I marker; Lanes 5-8, intestinal contents of control piglets. **(B)** Paraffin sections microscopy of duodenum, jejunum, and ileum tissues (100×). Different symbols in the pictures represented different pathological symptoms; yellow arrows represented congestion in the lamina propria, yellow boxes represented infiltration of inflammatory cells, and red boxes represented breakage and shedding of epithelial cells in the intestinal mucosa. **(C)** Porcine tissue expression distribution of *TFF1* in 11 tissues. **(D)** Differential expression of *TFF1* gene in duodenum, jejunum, and ileum. **(E)** Western blotting analysis of TFF1 protein level in jejunum. β-actin was used as internal reference proteins. **(F)** IPEC-J2 were infected with PEDV at MOI of 0.1. Cells were collected at 24, 48, and 72 hours post infection and detected mRNA expression of *TFF1* by RT-qPCR. Data are presented as mean values ± SD, using two tailed Student’s *t-test*. *p < 0.05, **p < 0.01. Each treatment has triplicate biological replicates at least.

The PCR products were amplified to detect PEDV, TGEV, PDCoV, PoRV in the small intestinal samples. The specific PCR products were only amplified in the infected samples with PEDV classic strain CV777 (**Figure 1A**). Our results show that PEDV is the unique infectious agent detected in diarrhea piglets (**Supplementary Figure 1A**). Histological analysis reveals that PEDV infection led to villi atrophy and partial shedding of villous epithelial cells (**Figure 1B**). The *TFF1* mRNA expression was shown at higher levels in the porcine duodenum, jejunum, and ileum (**Figure 1C**). To further validate the relationship between *TFF1* biological function and PEDV replication, we examined the mRNA expression of the *TFF1 in vitro* and *in vivo*, respectively. As shown in **Figure 1D**, the mRNA expression of *TFF1* in jejunum and ileum was significantly up-regulated in the PEDV group compared to that of control (*P* < 0.01), and TFF1 protein expression was also significantly increased in jejunum (*P* < 0.01, **Figure 1E**; **Supplementary Figure 1B**). Furthermore, the mRNA expression of *TFF1* was significantly up-regulated at 48 hours post infection of PEDV (*P* < 0.01, **Figure 1F**). These results provide evidence that *TFF1* is associated with the host responses toward PEDV infection.

### *TFF1* Is a Major Factor to Resist PEDV Replication

**Figure 2:**
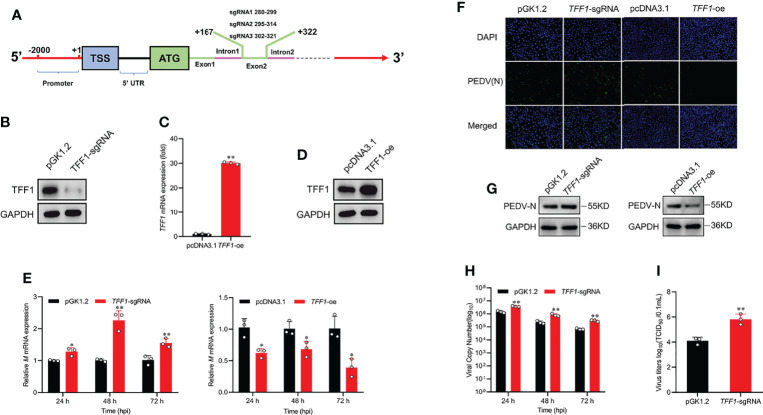
*TFF1* inhibits PEDV replication. **(A)** The location of the three sgRNAs (sgRNA1, sgRNA2, sgRNA3) used for *TFF1* knockout. **(B)** Western blot was performed to detect the knockdown efficiency of *TFF1*-sgRNA mixture pool cells compared with the pGK1.2 vector in IPEC-J2 cell lines. **(C, D)** Protein and mRNA levels of TFF1 were determined in IPEC-J2 transfected with *TFF1*-oe. **(E)** mRNA levels of *M* gene in IPEC-J2 transfected with *TFF1*-sgRNA and/or *TFF1*-oe after PEDV infection at MOI of 0.1 for 24, 48, and 72 hours. **(F, G)** The indirect immunofluorescence assay and western blotting were performed to detect PEDV (N) in PEDV-infected cells. *TFF1*-sgRNA and *TFF1*-oe cells were infected with PEDV at MOI of 0.1, respectively. **(H)** The PEDV genome copy number was measured by RT-qPCR. **(I)** Cell culture supernatants were collected at 24, 48, and 72 hours PEDV post infection and titrated for TCID50. Data are presented as mean values ± SD, using two tailed Student’s *t-test*. *p < 0.05, **p < 0.01. Each treatment has triplicate biological replicates at least.

To verify the function of *TFF1* during PEDV replication, we constructed *TFF1* knockout and overexpression IPEC-J2 cell lines (**Figures 2A–D**; **Supplementary Figures 2A–E**). We found that knockout of *TFF1* markedly up-regulated the mRNA expression of *M* gene, whereas *TFF1* overexpression down-regulated its expression upon PEDV infection (**Figure 2E**). The knockout also resulted in an enhanced viral particle, while *TFF1* overexpression led to a marked reduction of PEDV infectiousness (**Figures 2F, G**). Similarly, *TFF1* knockout significantly increased PEDV genome copy number (*P* < 0.01, **Figure 2H**). In addition, the cell culture supernatants were titrated for TCID_50_ and it reveals that the infectious titers were significantly enhanced in *TFF1*-knockout cells (*P* < 0.01, **Figure 2I**). Overall, these results suggest that the loss of *TFF1* expression exacerbates PEDV replication.

### *TFF1* Suppresses PEDV-Induced Jejunum Inflammation

**Figure 3:**
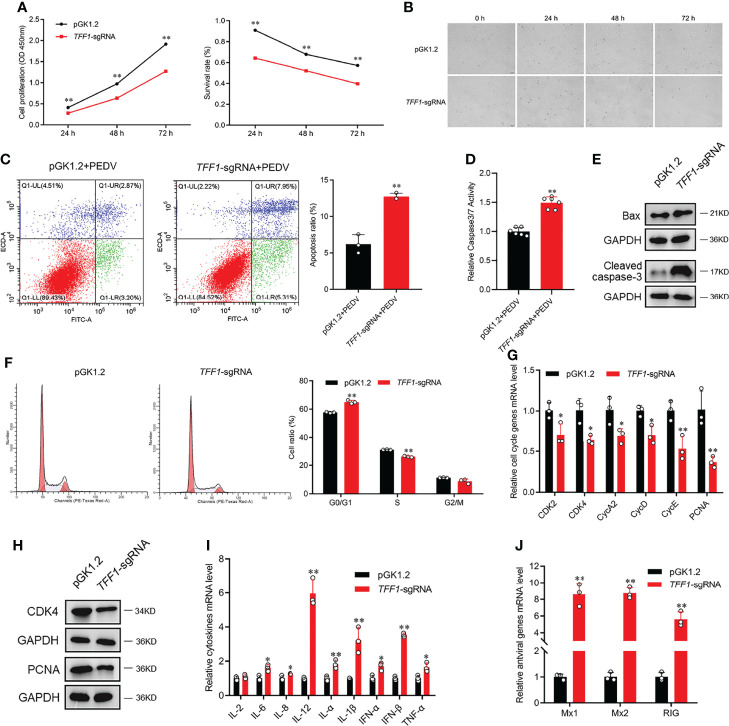
*TFF1* reduces PEDV-induced intestinal inflammation. **(A)** The proliferation and cytotoxicity of *TFF1*-knockout were detected by CCK-8 assay in IPEC-J2. **(B)** Morphological changes in *TFF1-*knockout cells after PEDV infection for 0, 24, 48 and 72 hours. **(C, D)** Cell apoptotic was detected by caspase3/7 activity and flow cytometry. **(E)** Protein levels of cell proliferation regulators were detected by western blotting in response to PEDV infection at MOI of 0.1 in *TFF1*-knockout cells. **(F)** Cell cycle distribution was detected by flow cytometry. **(G, H)** mRNA and protein levels of cell cycle genes were determined in *TFF1*-sgRNA cell. **(I, J)** mRNA expression of inflammatory cytokines and antiviral genes were detected after PEDV infection at MOI of 0.1 in *TFF1*-sgRNA cells. Data are presented as mean values ± SD, using two tailed Student’s *t-test*. *p < 0.05, **p < 0.01. Each treatment has triplicate biological replicates at least.

To investigate the effects of *TFF1* depletion on the growth and survival of IPEC-J2, we found that *TFF1* knockout decreased the viability of IPEC-J2 and promoted the development of cell lesion in a time-dependent manner (**Figures 3A, B**). Similarly, the knockout of *TFF1* also resulted in a poor survival of IPEC-J2 as measured by CCK8 and caused the pronounced apoptosis after PEDV infection, as reflected by the activation of caspase3/7 with flow cytometry analysis (*P* < 0.01, **Figures 3C, D**). Moreover, *TFF1* knockout led to the significantly decreased - proteins which are important for cell proliferation, growth, and survival, along with an increased levels of Bax and Cleaved-Caspase 3 (**Figure 3E**). Additionally, we found that *TFF1* knockout reduced the S-phase but increased the cell growth in the G0/G1 phase (**Figure 3F**). The expression of mRNA and protein for cell cycle genes were significantly down-regulated (**Figures 3G, H**). Furthermore, the mRNA expression of proinflammatory cytokines and antiviral genes were significantly up-regulated after *TFF1* knockout (**Figures 3I, J**). These results indicate that *TFF1* depletion alters the cell cycle and exacerbates PEDV-induced cell death and inflammatory responses.

### DNA Methylation Contributes to TFF1 Expression During PEDV Infection

**Figure 4:**
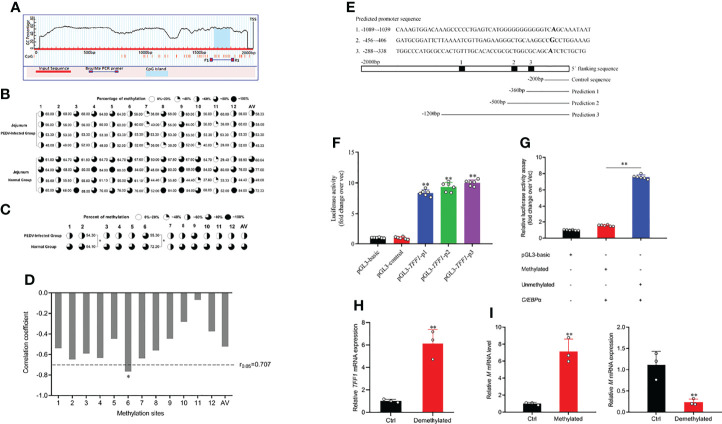
mC-6 methylation is involved in C/EBPα-mediated *TFF1*. **(A)** CpG island prediction of porcine *TFF1* upstream 2000 bp promoter region. The long red bar represents the promoter sequence; dark blue line shows designed methylation primer; blue bar indicates the CpG island, TSS means transcriptional start site. **(B, C)** Bisulfite genomic sequencing analysis of methylation profile in 12 CpG sites and average methylation status in CpG island of the *TFF1* promoter. AV, average methylation status. **(D)** The correlation analysis between methylation status of each CpG site and mRNA expression of *TFF1* in jejunum. **(E)** Prediction of *TFF1* core promoter region and amplification of truncated fragments. **(F)** Identification of core promoter region of *TFF1* by four different truncated plasmids. **(G)** The relative fluorescence intensity of *TFF1* was determined by luciferase reporter assay in vectors treated with or without methyl transferase *M.Sss*I. IPEC-J2 were co-transfected with *C/EBPα*-oe and methylated or unmethylated vectors. **(H)** Relative mRNA expression of *TFF1* was determined by RT-qPCR in IPEC-J2 treated with or without 0.1 μM 5’-Aza-2’-dC for 72 hours. **(I)** Relative mRNA expression of *M* gene was determined by RT-qPCR in IPEC-J2 treated with or without *M.Sss*I after PEDV infection at MOI of 0.1. Data are presented as mean values ± SD, using two tailed Student’s *t-test* analysis. *p < 0.05, **p < 0.01. Each treatment has triplicate biological replicates at least.

The CpG islands (located at −477 to −299 bp) were predicted by MethPrimer (**Figure 4A**; **Supplementary Figure 3A**). As shown in **Figure 4B**, bisulfite**-**sequencing results reveal that there was different methylation status of 12 CpG sites. Methylation analysis demonstrates that mC-2 loci and mC-6 loci presented hypomethylation status compared to that of the control (*P* < 0.05, **Figure 4C**). Pearson correlation analysis shows that there was a significantly negative correlation between methylation status and *TFF1* mRNA expression in the mC-6 loci (R =−0.78, r_0.05_ = 0.707, *P* =0.022, **Figure 4D**). According to the predicted core promoter sequences, we constructed four truncated recombinant vectors (**Figure 4E**; **Supplementary Figure 3B**). As shown in **Figure 4F**, luciferase reporter assay shows that the transcriptional activity of pGL3-*TFF1*-p1 was remarkedly higher than that of pGL3-control (*P* < 0.01), indicating that the core promoter region of *TFF1* was located at −360 to −200 bp. To investigate the epigenetic mechanisms of *TFF1* regulation, we first generated a methylated vector DNA substrate with the methyl transferase of *M.Sss*I. Then, we transiently transfected the *C/EBP*α overexpression vector that failed to activate methylated reporter of *TFF1* compared to the unmethylated reporter (**Figure 4G**). However, the mRNA expression of *TFF1* was markedly upregulated after 5’-Aza-2’-dC treatment compared to that of control, indicating that DNA methylation inhibition strongly enhanced gene expression of *TFF1* (**Figure 4H**). Furthermore, *M.Sss*I or 5’-Aza-2’-dC treatment before PEDV infection considerably regulated PEDV entry in IPEC-J2. As shown in **Figure 4I**, PEDV invasion was significantly inhibited by 5’-Aza-2’-dC, while the expression of *M* gene was significantly upregulated with *M.Sss*I treatment. These results suggest that the hypermethylation of *TFF1* facilitates PEDV replication.

### *C/EBPα* Acts as a Key Modulator to Activate- *TFF1* Gene Expression

**Figure 5:**
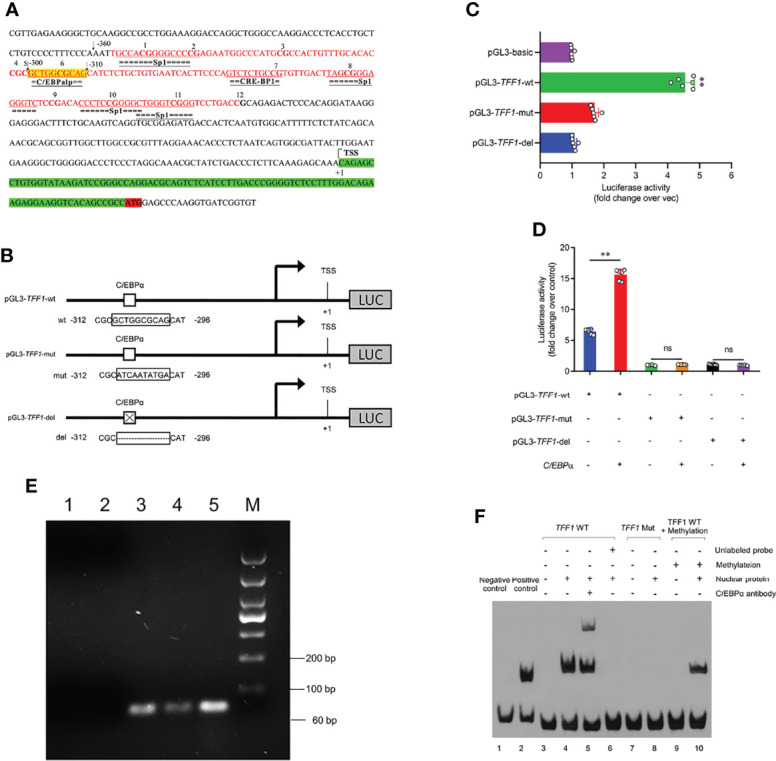
*C/EBP*α activates the *TFF1* expression. **(A)** Schematic representation of the *TFF1* promoter sequences. *C/EBP*α potential binding site is highlighted in yellow. CpG island is highlighted in red and CG sites are shown in bold font. TSS was the transcription start site, and defined as +1. **(B, D)** The pGL3-*TFF1* recombinant vectors (with or without *C/EBP*α consensus site) were constructed and co-transfected with *C/EBP*α-oe into IPEC-J2. **(C)** The pGL3-*TFF1* constructs (pGL3-*TFF1*-wt, pGL3-*TFF1*-mut and pGL3-*TFF1*-del) were transfected into IPEC-J2. Luciferase reporter assays were performed 36 hours post transfection. **(E)** Identification of *C/EBP*α binding to *TFF1* promoter by ChIP-PCR. Lane 1, PCR amplification with RNase-free H_2_O (blank control); Lane 2, PCR amplification with Rabbit IgG antibody (negative control); Lane 3, PCR amplification with anti-C/EBPα antibody; Lane 4, PCR amplification with RNA Polymerase II antibody (positive control); Lane 5, PCR amplification with Input DNA; Lane M, DL1000 DNA marker. **(F)** EMSAs confirmed the binding of the C/EBPα protein to the *TFF1* promoter region. All free biotin-labeled probes (*TFF1* WT probes, Lane3-6, and Lane9-10; *TFF1* Mut probes, Lane7-8) were examined by streptavidin-HRP conjugate. Lane 5, Super-shift mobility band was confirmed complex of biotin-labeled *TFF1* WT probes and C/EBPα protein. Lane 6, unlabeled probes was a cold competitor probe. Lane 9-10, *TFF1* WT probes were treated by methyl transferase *M.Sss*I. Data are presented as mean values ± SD, using two tailed Student's t-test analysis. **P < 0.01, ns, not significant. Each treatment has triplicate biological replicates at least.

Having demonstrated the crucial role of *TFF1* in IPEC-J2, we next examined whether *C/EBP*α participated in the transcriptional regulation of *TFF1*. As shown in **Figure 5A**, we further predicted the potential transcription factors in 12 CpG sites of *TFF1* promoter, in which mC-6 loci was found to be within the binding domain of *C/EBP*α. We then performed the reporter-gene assays with the promoter of *TFF1* and found that it was highly responsive to *C/EBP*α-mediated transactivation. The mutation or deletion of the putative *C/EBP*α binding site effectively diminished the *C/EBP*α-dependent activation (**Figures 5B, C**). Using transient co-transfection and reporter gene assays, we found that overexpression of *C/EBP*α resulted in transactivation of the *TFF1* gene promoter, suggesting that *C/EBPα* can act as an activator of *TFF1* (**Figure 5D**). Finally, a positive DNA fragment was detectable in the products amplified with chromatin fragments that were precipitated using specific anti-C/EBPα antibody (**Figure 5E**). Together, the data indicate that *C/EBP*α activates TFF1 target promoters *via* the putative *C/EBP*α binding site. To investigate the influence of DNA methylation on the nuclear protein binding properties, we performed EMSA using 5′-biotinylated probes corresponding to putative *TFF1* sequences including the mC-6 loci. Specifically, two different double-stranded oligonucleotides (wild or mutant type) were treated with or without methyl transferase *M.Sss*I and incubated with nuclear extracts from IPEC-J2. Thereafter, the wild-type probes resulted in significantly increased mobility shift of nuclear proteins compared with the mutant-type and methylated wild-type probes. The shift can be competed by an excess of unlabeled 5′-biotinylated probes which restrain *C/EBP*α binding to *TFF1*. Additionally, the specific binding of C/EBPα to the *TFF1* was further confirmed using an anti-C/EBPα antibody in a super-shift assay (**Figure 5F**). These results show that methylation at mC-6 loci blocks the C/EBPα binding to *TFF1* promoter region.

### Enhanced *C/EBPα* Exhibited Antiviral Activity

**Figure 6:**
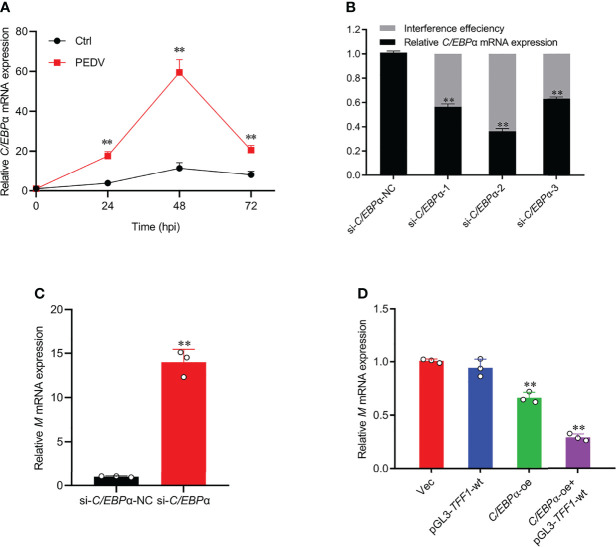
*C/EBP*α exhibited antiviral activity. **(A)** IPEC-J2 were infected with PEDV at MOI of 0.1. Cells were collected at 24, 48, and 72 hours post infection and detected mRNA expression of *C/EBPα* by RT-qPCR. **(B)** Knockdown efficiency of si-*C/EBP*α-1, si-*C/EBP*α-2, and si-*C/EBP*α-3 was detected by RT-qPCR. **(C)** Relative mRNA expression of *M* gene was determined by RT-qPCR in IPEC-J2 transfected with si-*C/EBP*α and si-*C/EBP*α-NC. **(D)** Relative mRNA expression of *M* gene was determined by RT-qPCR in IPEC-J2 co-transfected with pGL3-*TFF1*-wt and *C/EBP*α-oe after PEDV infection. Data are presented as means ± SD, using two tailed Student’s t-test analysis. **p < 0.01. Each treatment has triplicate biological replicates at least.

To examine the regulatory role of *C/EBP*α in PEDV infection, the cells with PEDV treatment resulted in a similar up-regulation of *C/EBP*α with that of *TFF1* gene (**Figure 6A**). Therefore, siRNAs of *C/EBP*α were transfected into IPEC-J2 (**Figure 6B** and **Supplementary Figure 4A**). Interestingly, *C/EBP*α silencing played an important role in the viral replication, exhibiting that mRNA expression of *M* gene was significantly up-regulated (**Figure 6C**). Subsequently, *C/EBP*α overexpression vector was constructed (**Supplementary Figures 4B, C**) and co-transfected with promoter region of *TFF1* significantly reduced the invasion rates of PEDV (**Figure 6D**), indicating that the *C/EBP*α plays an effective role in antiviral target activator on regulating *TFF1* expression.

## Discussion

The intestinal barrier integrity is essential for the maintenance of homeostasis, while its dysfunction causes significant consequences and thereby gastrointestinal diseases, such as viral and bacterial infection. PEDV strains have been characterized by rapid proliferation in and destruction of intestinal epithelial cells (IECs) among suckling piglets (28) but milder in adult pigs (29, 30). A variety of viruses was considered a leading cause of severe gastroenteritis and diarrhea (31, 32), determined by the process of intestinal development in young animals. There were vacuolated small intestinal epithelial cells in most 2-week-old pigs but hardly detected after 3-4 weeks from the newborn (33). The turnover of the small intestinal epithelium in the newborns (7-10 days) was slower than that of the weaned piglets (2-4 days) (34). The blunted recovery may result from the susceptibility to various infections like PEDV at the newborn stage. Thus, it is vital to determine the PEDV and host mucosa interactions and the onset of the disease. Our results and others have demonstrated that *TFF1* functions to restore and maintain the gastrointestinal mucosal barrier integrity (35, 36). While TFF1 abnormal expression, together with the characteristics of diarrhea population is subjected to intestinal villous atrophy, deepening crypts depth in the PEDV-infected suckling piglets. The specific responses of TFF1 hyper-expression have been revealed by Ren (37) and Soutto (38), who found pathologically increased *TFF1* expression in gastroenteritis and gastrointestinal ulcers. In previous studies, a significant up-regulation of *TFF1* expression after PEDV infection has been proved (39). Herein, we addressed that *TFF1* was highly expressed in the jejunum in response to PEDV and revealed it as the primary loci, where *TFF1* started to defend further PEDV entry by the enhanced transcription. Indeed, *TFF1* knockout mouse has been generated previously and exhibited pro-inflammatory responses in the small intestine with an abnormal mucosal epithelium proliferation and an obvious impaired barrier function (40). Being consistent with the actions of *TFF1* deletion, the knockout of the *TFF1* gene in IPEC-J2 significantly suppresses cell proliferation and induces apoptosis. In our model, there is also an enhanced viral particle, copy number, and virus titer of PEDV. In this regard, as we assumed that *TFF1* is one of the key factors to resist the PEDV infection and the ectopic expression of TFF1 strongly inhibited PEDV replication. Notably, TFF1 functions enrolled in cell growth and early differentiation contribute to its protective role probably through proliferation-associated β-catenin signaling pathway (41). Thus, we provided evidence for supporting *TFF1*-controlled cell cycle and arrest. Our evidence exhibited that a significantly increased cell proportion at the G1 phase, with a drastic reduction at S phase, was caused by *TFF1* knockout. According to these clues of cell proliferation by *TFF1* depletion during PEDV invasion, we suggest that *TFF1* possibly re-establishes the epithelium community *via* promoting cell proliferation. It is well-known that the viral infection-triggers severe inflammatory responses as one of the main events of pathogenesis (42). Importantly, IECs integrate signals to modulate inflammatory responses and facilitate intestinal homeostasis as reported (43). It is reasonable that couples of inflammatory cytokines were secreted which helps to prevent viral entry of the gut barrier (44). These responses have also been displayed in PEDV infection. Herein, we showed the altered RIG-I signaling pathway, RIG-I-like receptors, and Mx1 and Mx2 in infected intestine. The selections are critical for anti-viral actions in IECs (45, 46), inducing IFN-I (*IFN-*α/*IFN-*β) production and also working for the host innate immune system when confronted with the viral invasion (47). Given the fact that the pro-inflammatory cytokines, such as TNF-α and interleukins, are activated by the innate immune system, we found that the cytokines associated with antiviral genes were all elevated. This suggests that innate immune responses functioned through cytokines and are involved in the PEDV-infection for the defense responses.

The transcription factor CCAAT enhancer binding protein α (C/EBPα) is essential for normal cell development and evidence suggests that impaired function resulting from silencing *C/EBP*α contributes directly to disease development (48). In agreement with a previous study, we also verified that *C/EBP*α knockdown was significantly associated with enhanced PEDV virulence. Furthermore, C/EBPα is involved in various innate immune functions such as inflammatory cytokines production and immune response against HIV infection by TLR3-mediated (49). Therefore, up-regulated *C/EBP*α expressions may prime the cells for generating a robust immune response against PEDV infection. C/EBPβ would prime cell differentiation and promote the expression of *TFF1* by inducing *C/EBPα* expression *in vivo*. In parallel, *TFF1* expression is upregulated in C/EBPα-transfected cells *in vitro* (24). Activating TFF1 production, an important event in PEDV infection suppression, could follow the overexpression of *C/EBPα* and/or promoter hypomethylation. Abnormal alterations of methylation status are always associated with the transcription silencing and contribute to disease development (50). By preventing the binding of transcription factors to their motifs, DNA methylation can therefore block transcription activation of gene promoters (51). In previous studies, the epigenetic regulation modulated *TFF1* expression has been proved (52, 53), whereas the epigenetic modifications of *TFF1* function under PEDV infection is still unknown. As we assumed, PEDV changed the DNA methylation profiles in which hypomethylation of mC-6 loci was observed to in association with *TFF1* expression. Being consistent with the actions of mC-6 loci methylated, *TFF1* gene transcriptional activity was reduced. Notably, the mC-6 loci containing the *C/EBP*α binding domain was also located in the *TFF1* core promoter region. It is worth mentioning that TFF1 transcription was highly responsive to *C/EBP*α-mediated transactivation and, therefore, plays a crucial role in the activation of gene transcription, cell proliferation, and differentiation (54). In agreement with the results of Gonçalo Regalo et al. (24), we verified that C/EBPα can bind to the putative motif in porcine *TFF1* promoter and perform its transactivated action. This is associated with the finding of C/EBPα-medicated *TFF1* by inhibiting the Ras/MAPK pathway due to their co-localization (24). Indeed, Ras/MAPK signaling is heavily involved in C/EBPα-dependent regulation of specific antivirus genes, which may be an alternative way to modify *TFF1* expression during PEDV infection and need to be further validated.

**Figure 7:**
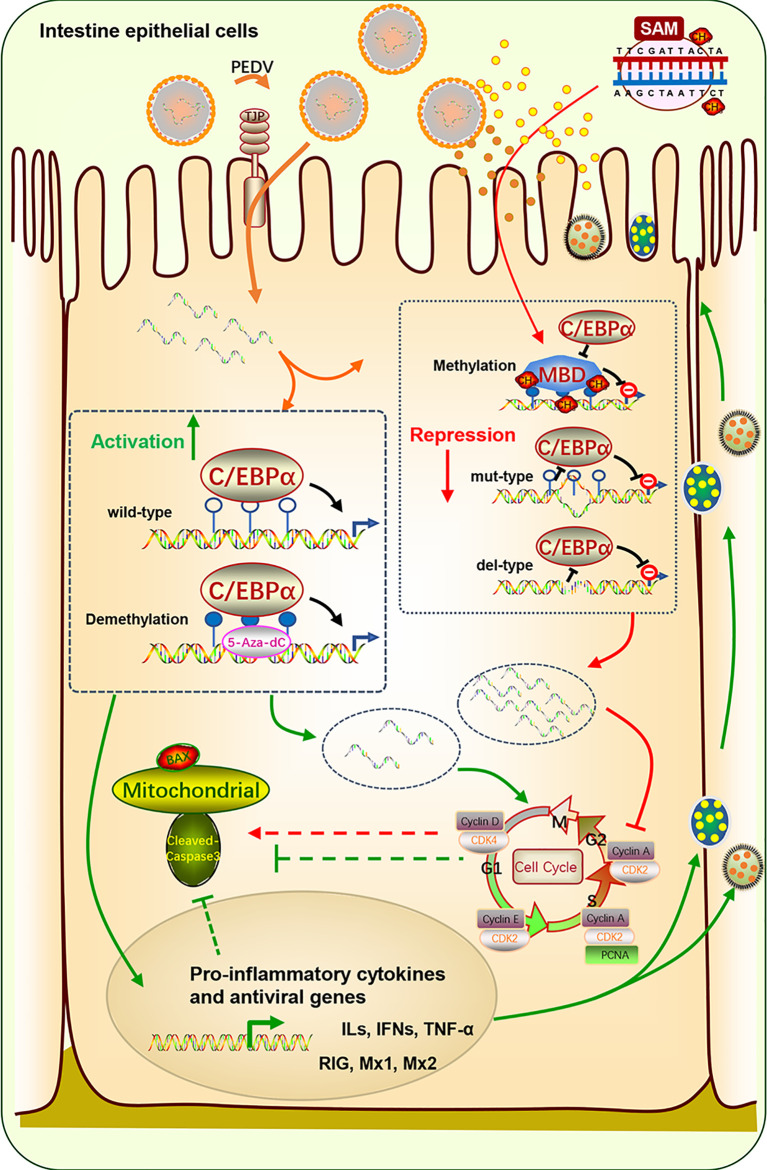
Schematic diagram of C/EBPα-modulated TFF1 *via* mC-6 methylation in the PEDV-induced jejunum inflammation. *TFF1* is highly expressed in intestinal epithelium during PEDV-infected jejunum inflammation. It improves the intestinal functions through reducing pro-inflammatory cytokines and facilitating the inhibition of the virus replication by increasing antivirus genes. This process further benefits the porcine intestinal epithelium cell proliferation and growth by inhibiting apoptotic kinases. The mucous repairment is therefore help the host resistant to the porcine *coronavirus* infection. Mechanistically, this virus affects DNA methylation profile especially the mC-6 loci methylation including the key factor C/EBPα binding sites. The transformation alteration of C/EBPα is detrimental for the specific bind to *TFF1*. This process determinates the *TFF1* action of jejunum inflammatory responses suffer from PEDV invasion.

A graphic illustration of the C/EBPα-mediated epigenetic regulation of *TFF1* in PEDV infection is shown in **Figure 7**. This study indicated that *TFF1* is highly expressed in the inflamed jejunum epithelium during PEDV-infection. The *TFF1* expression improves the intestinal functions through down-regulating pro-inflammatory cytokines gene expression like *IFNs* and *TNF-α*, and facilitate the inhibition of the virus replication by increasing antivirus genes likes *RIG*, *Mx1* and *Mx2*. This process further benefits the porcine intestinal epithelial cell proliferation and growth by inhibiting apoptotic kinases such as Bax and Caspase 3. Therefore, the repairment helps the host to fight against the porcine *coronavirus* infection. Mechanistically, this virus affects the DNA methylation profile, especially the mC-6 loci methylation, including the key factor C/EBPα binding sites. The transformation alteration of C/EBPα is detrimental to the specific bind to *TFF1*. This process drives the *TFF1* action of jejunum inflammatory responses when suffering from PEDV invasion.

In this study, we confirmed that high expression of *TFF1* contributes to enhancing the host resistance to PEDV invasion. Additional, upregulated of *C/EBP*α expression also partially inhibited PEDV replication. Activating TFF1 production could follow the overexpression of *C/EBP*α and/or promoter hypomethylation, which contributes to enhancing piglets’ resistance to PEDV. Collectively, our results feature a novel connection between *TFF1* and regulator C/EBPα for PEDV resistance in porcine intestinal epithelium. Our discoveries provide unique insights into the potential modulation of DNA methylation to mediate the *C/EBP*α-dependent *TFF1*. Our findings contribute to the field of intestinal protection, anti-inflammation, and anti-coronavirus in porcine and other species. Challenges remain as to address complex mechanisms of *coronaviruses* and *TFF1* can be regarded as a potential biomarker candidate and therapy target for further studies.

## Data Availability Statement

The original contributions presented in the study are included in the article/**Supplementary Material**. Further inquiries can be directed to the corresponding authors.

## Ethics Statement

The animal study proposal was approved by the Institutional Animal Care and Use Committee (IACUC) of the Yangzhou University Animal Experiments Ethics Committee (permit number: SYXK (Su) IACUC 2012-0029, Approval Date: 09-12-2012). All experimental methods were conducted in accordance with the relevant guidelines and regulations for the Administration of Affairs Concerning Experimental Animals approved by the State Council of the People’s Republic of China.

## Author Contributions

HQ and QZ performed most of the experiments; HW and SW participated in the experiments; WB and DC conceived the study, HQ, QZ, and DC participated in its design and coordination, and wrote the manuscript. All authors read and approved the final manuscript.

## Funding

This study was supported by the National Natural Science Foundation of China (No. 31972535), the Jiangsu Agricultural Science And Technology Innovation Fund [CX(21)2014], the Science and Technology Support Program of Jiangsu Province (BE2019344) and the Priority Academic Program Development of Jiangsu Higher Education Institutions.

## Conflict of Interest

The authors declare that the research was conducted in the absence of any commercial or financial relationships that could be construed as a potential conflict of interest.

## Publisher’s Note

All claims expressed in this article are solely those of the authors and do not necessarily represent those of their affiliated organizations, or those of the publisher, the editors and the reviewers. Any product that may be evaluated in this article, or claim that may be made by its manufacturer, is not guaranteed or endorsed by the publisher.
