# Supplementary material for: C/EBPα Epigenetically Modulates TFF1 Expression via mC-6 Methylation in the Jejunum Inflammation Induced by a Porcine Coronavirus
Source: Front Immunol. 2022 May 25;13:881289. doi: 10.3389/fimmu.2022.881289 (PMC9174463; doi:10.3389/fimmu.2022.881289)
Supplement: Supplementary file 1 [file DataSheet_1.docx]

Supplementary Material

## Supplementary Figures


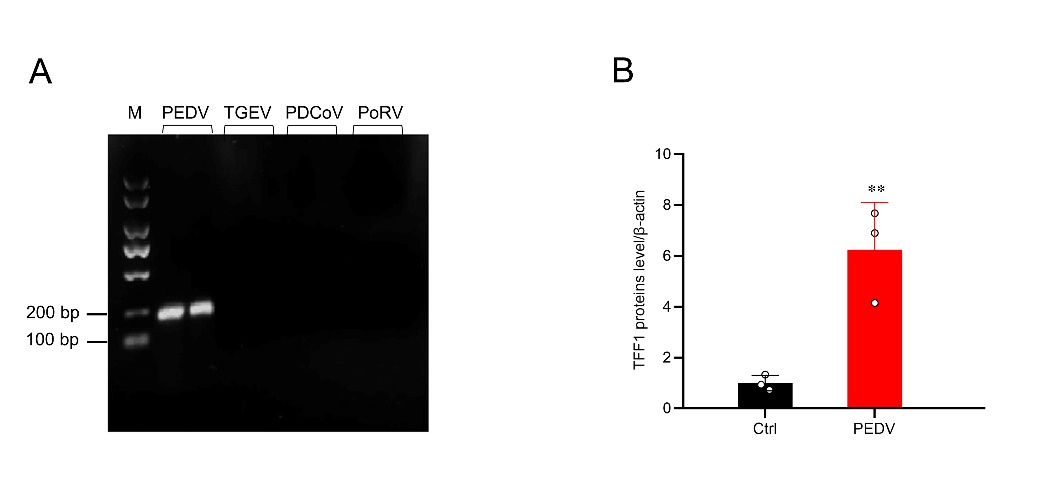


**Supplementary Figure 1.** **Pathogen identification of diarrhea piglets.** (A) Etiology identification of multiple porcine coronaviruses. Lane2-3, PEDV, Lane 4-5, TGEV, Lane 6-7, PDCoV, Lane 8-9 PoRV, Lane M, DL1000 DNA marker. (B) Quantification analysis of TFF1 protein to β-actin in jejunum. Data were presented as mean values ± SD, using two tailed Student's *t-test*. **, *p* < 0.01. Each treatment has triplicate biological replicates at least.


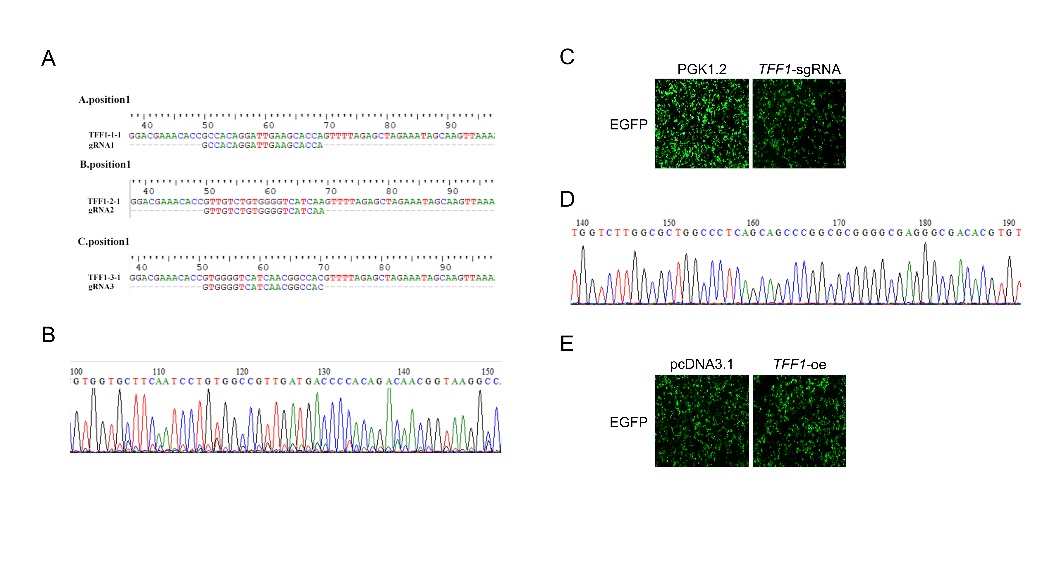


**Supplementary Figure 2.** **Construction of *TFF1* knockout and overexpression plasmid.** (A) Colony PCR verification of the *TFF1*-sgRNAs (sg1, sg2, and sg3) plasmids by sequencing. (B) The identification of knockout efficiency in *TFF1-*sgRNA pool cells by PCR sequencing. (C) Fluorescence expression efficiency of *TFF1*-sgRNA in IPEC-J2 cells. (D) The identification of overexpression plasmid constructs in *TFF1-*oe pool cells by PCR sequencing. (E) Fluorescence expression efficiency of *TFF1*-oe in IPEC-J2 cells.


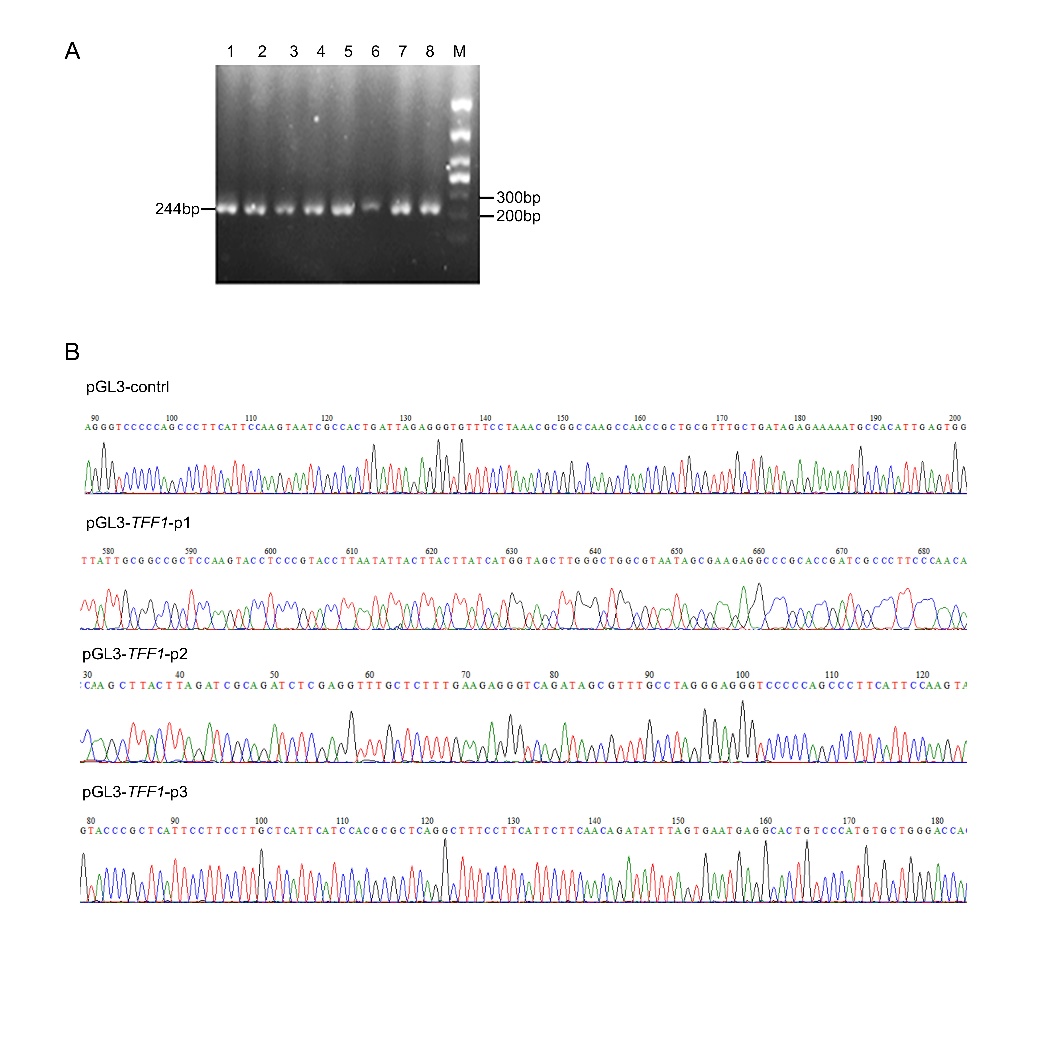


**Supplementary Figure 3. PCR amplification of CpG island and core promoter of *TFF1* promoter.** (A) Bisulfite conversed DNA was performed by PCR amplification in jejunum of diarrhea and normal piglets. Lane 1-4, diarrhea piglets; Lane 5-8, normal piglets; Lane M, DL1000 DNA marker. (B) PCR sequencing results of the truncated plasmids (pGL3-control, pGL3-*TFF1*-p1, pGL3-*TFF1*-p2, pGL3-*TFF1*-p3).


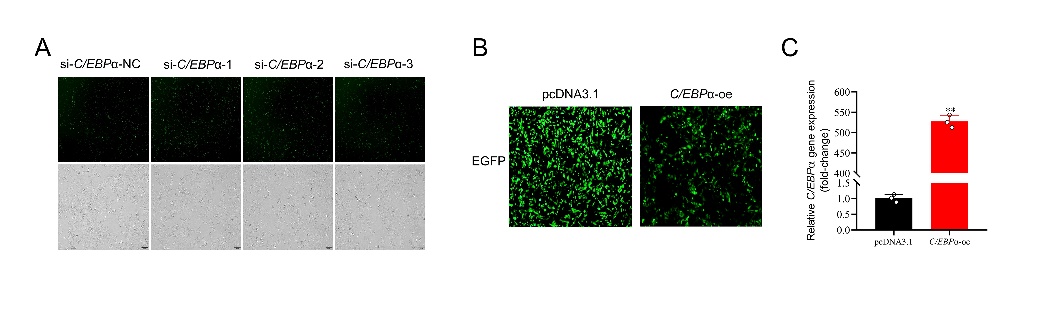


**Supplementary** **Figure 4.** **Efficiency verification of *C/EBP*α knockdown and overexpression.** (A) The fluorescence and white imaging of four *C/EBP*α-interfering siRNAs transfected into IPEC-J2 cells under microscope (100×). (B) Fluorescence expression efficiency of *C/EBP*α-oe in IPEC-J2 cells. (C) The efficiency detection of *C/EBP*α overexpression by RT-qPCR analysis. Data was shown as mean values± SD, using two tailed Student's *t-test*. **, *p* < 0.01. Each treatment has triplicate biological replicates at least.
